# Molds and the lungs

**DOI:** 10.4103/0970-2113.71937

**Published:** 2010

**Authors:** Bharat Bhushan Sharma

**Affiliations:** *Deparment of Medicine, SMS Medical College, Jaipur, India. E-mail: drbbshar08@yahoo.co.in*

Molds are ubiquitous and they are integral parts of flora and fauna of the world. They are small microorganisms of varied size but with a great capacity to form reproductive elements. Respiratory physicians are very well aware of their potential of producing allergic and non-allergic lung diseases. Lung alveoli and the bronchi along with the upper respiratory tract are open to external environment. This provides a favorable biomaterial for the growth of microorganisms like molds, especially if the defense mechanisms are compromised.

Lung defense mechanisms usually suppress the growth of the microorganisms but the fungi have some unique properties that favor their growth. Defense mechanisms of lung include natural innate immunity, anatomical barriers like cilia and lung surfactant. Fungal virulence factors that try to counteract the defenses are many and include various enzymes, mainly proteolytic in nature, which are better used by microorganisms to produce invasive forms of fungal diseases. Expressions of these factors also depend upon type of environment in which growth of fungus occurs.[1]

Thus, depending upon the host environment and virulence properties of the fungus, a particular respiratory ailment is produced. The mold-related illnesses can be broadly divided into infections and immune/allergies. Immune/allergic diseases are more common than infectious illnesses. Environmental exposure is the most common mode of contraction of the diseases related to molds. It will be quite appealing to divide patients into different environmental categories, for example, farming versus non-farming community. Farmers are significantly more predisposed to a variety of fungal agents. An interesting study showed that agricultural dust in India represents an occupational hazard as it contains high concentrations of allergenic microorganisms especially molds.[2]

Molds can be sampled from environment by various techniques depending upon availability and expertise. Tape-lift, bulk samples from carpets, soil culture and open plate culture are some of the useful techniques. Spore count can be better done with the help of air samplers. Serological studies, including precipitins, usually IgG antibodies, represent some of the important tools in diagnosis. Recovery of organism in tissue sample is the best evidence, while airway colonization may represent a contamination. Some of the fungal diseases produce typical radiographic patterns which could be useful in diagnosis of that condition eg, central bronchiectasis in allergic bronchopulmonary mycosis (ABPM).

Aspergillus species is particularly important as it commonly affects both immuno-compromised and immuno-competent host lungs. The main illnesses caused by aspergillus include-1) As allergen in bronchial asthma, 2) ABPM, 3) Hypersensitivity pneumonitis, 4) Aspergilloma, 5) Invasive aspergillosis, 6) Commensal in sputum.[3] The conditions other than invasive aspergillosis usually occur in immuno-competent hosts. Aspergillus can grow well in both hot and cold climates and produce a large number of spores which are asexual reproductive structures. Temperature range as low as 15°C or as high as 55°C or even more may support the growth of this species.[1] Aspergillus spores have been detected in almost all environmental setups which may be moldy hay, decaying vegetables, wood chips or even bird droppings. A fumigatus has some of the features which make it more pathogenic to humans. These features are small spore size which can aerosolize effectively and easily reach lung alveoli, relative resistance to oxidative killing, and survival at wide range of temperatures.[4] Moreover, a high concentration of spores has been linked to ABPM[5] and has been associated with exacerbations.

One interesting point that emerges out of asthma and mold relationship is that asthma may result from a specific response against fungal antigen that could restrict the organism to the airway surface, thereby preventing invasion. That is why immunosuppressive therapies like corticosteroids are effective in these patients.[6]

Aspergillus genus is composed of many pathogenic species like A. fumigatus, A. flavus, and A. parasiticus. Aflatoxins are produced by A. flavus and parasiticus while A. fumigatus usually has not been reported to produce potent toxins. ABPM is mainly caused by A. fumigatus, other fungi that can cause ABPM include- A. nidulans, A. niger, A oryzae, A terreus. A flavus and A ochraceous. ABPM is relatively common in India; however, it is usually under diagnosed. High index of suspicion for diagnosis of ABPM is to be kept because timely intervention may reduce unnecessary morbidity and at times mortality.[7]

The prefix ‘Myco’ in Mycobacterium tuberculosis is a misnomer as this bacterium is not biologically linked to fungus. However, fungal ball like aspergilloma occurs at increased frequency in these patients. Occurrence of such ball like lesions has also been described with cystic fibrosis and sarcoidosis. The exact pathogenetic mechanism which could favor formation of this interesting entity is not yet clear. This is a fascinating subject for research which could further highlight mold lung interactions. Aspergilloma may also occur with ABPM emphasizing inter-relation of the different manifestations of fungal infections.[8]

Interestingly, this issue of lung India incorporates two interesting studies one from North and another from East of India.[9,10] These studies describe the characteristics of patients with Aspergilloma and ABPM, two forms of aspergillosis usually seen in immuno-competent hosts.

Gupta *et al*.,[9] in their study, re-emphasized the notion that the clinical profile of pulmonary aspergilloma is quite variable. Almost half of the patients presented with hemoptysis, and rest with other chest symptoms. A good number of patients had clinical symptoms suggestive of chronic necrotizing pulmonary aspergillosis. Some patients even had clinical symptoms suggestive of ABPM. This again shows varied nature of presentation of aspergillus associated pulmonary involvement.

Sarkar *et al*.,[10] conclude that a careful search for ABPM should be done in all the cases of mold-sensitive asthma. One third of their asthmatic patients were sensitive to mold. Quarter of these sensitive patients had ABPM making an overall incidence in asthmatic patients to be approximately 8%. In standard western literature, its’ incidence is around2%.[1] Variable occurrences reported in some of the studies are probably because of higher center referral bias and lack of uniform criteria to diagnose the patients. Most of the patients in this study were in category III (exacerbation) and IV (corticosteroid dependent asthma) of ABPM. Similarly, FEV1 was also lower in these patients as compared to patients without ABPM. One of the most important points to be noted is that the increased awareness of this potentially disabling disease can help in early diagnosis and institution of appropriate therapy to prevent further lung damage.

Last year, 7,364 articles on aspergillus were indexed in PubMed but still, a lot is left to be learnt about various aspects of pathogenesis mechanisms, best diagnostic and treatment modalities for mold- related lung involvement. There is a great potential of research in this field in the future.
